# Diagnostic value of contrast-enhanced CT for elbow joint disorders in dogs

**DOI:** 10.3389/fvets.2025.1626472

**Published:** 2025-10-16

**Authors:** Bettina Rohr, Sven Reese, Martin Zöllner, Andrea Meyer-Lindenberg

**Affiliations:** ^1^Clinic for Small Animal Surgery and Reproduction, Centre for Clinical Veterinary Medicine, LMU Munich, Munich, Germany; ^2^Institute of Veterinary Anatomy, Histology and Embryology, LMU Munich, Munich, Germany

**Keywords:** contrast-enhanced CT, elbow disorders, dog, elbow dysplasia, flexorenthesopathy

## Abstract

**Objective:**

Computed tomography (CT) with intravenous contrast agents can provide additional diagnostic information. To our knowledge, no previous study has evaluated the role of contrast agents in CT imaging of the canine elbow joint. This study aimed to determine the diagnostic value of post-contrast imaging.

**Methods:**

A total of 326 elbow joints from 163 dogs with unilateral or bilateral lameness caused by elbow joint pathology were examined using radiography and CT, both with and without contrast agents. Diagnoses assessed from radiographs and CT scans in the bone window included primary diseases and periarticular osteophytes. Possible primary diseases included medial coronoid process disease (MCPD), ununited anconeal process (UAP), osteochondrosis dissecans (OCD), humeral intracondylar fissure/incomplete ossification of the humeral condyle (HIF/IOHC), epicondylar spur, caudal or medial calcified bodies. Contrast enhancement of the joint capsule and flexor muscles was evaluated in the soft tissue window. Elbow joints without pathological findings, lameness, or contrast enhancement served as controls (*n* = 21).

**Results:**

Among the 137 elbow joints showing contrast enhancement, 94 demonstrated enhancement limited to the joint capsule, 16 to the flexor muscles, and 27 to both joint structures. Epicondylar spurs were most strongly associated with flexor muscle enhancement, whereas medially located calcified bodies were not. Joint capsule enhancement was most frequently associated with periarticular osteophytes. Notably, elbows with flexor muscle involvement but no epicondylar spur or calcified bodies (*n* = 14) could only be detected through contrast-enhanced imaging.

**Conclusion:**

A contrast agent could be administered during most CT scans of the elbow joint to ensure that no pathological changes are overlooked. Nevertheless, it is important to weigh the benefits for treatment and prognosis against the risks of administering a contrast agent to the patient.

## 1 Introduction

Forelimb lameness due to elbow joint disorders is a common reason for dogs to be presented to a veterinary orthopedic practice. Elbow joint disorders are frequently observed in growing dogs, regardless of the underlying cause (1). Medium-sized dog breeds are particularly predisposed to elbow dysplasia (ED), a disease complex that encompasses osteochondrosis dissecans (OCD), ununited anconeal process (UAP), medial coronoid process disease (MCPD), and elbow incongruence (2). Conditions within the ED complex may occur individually or in combination (3–6). MCPD encompasses osteocartilaginous fragments, fissures, or abrasions of the cartilage and subchondral bone of the medial coronoid process.

The humeral intracondylar fissure (HIF), formerly termed incomplete ossification of the humeral condyle (IOHC), is another elbow joint disorder that may cause forelimb lameness (7). HIF/IOHC is characterized by a sagittal fissure in the humeral condyle that partially or completely separates the two halves of the distal humeral condyle (7–9). HIF is regarded as a stress fracture of the humeral condyle or results from failure of union at the intracondylar growth plate (IOHC) (7–9).

Flexor enthesopathy is also recognized as a cause of elbow pain in medium and large breeds (10). This condition involves the flexor muscles and their attachment to the medial epicondyle (11–13). The term encompasses all lesions affecting the medial epicondyle of the humerus, regardless of cause (14). Reported conditions include an ununited medial epicondyle, persistence of a preformed ossification center, traumatic avulsion of the medial humeral epicondyle, dystrophic calcification at the origins of the flexor tendons, and spur formation at the caudal aspect of the medial epicondyle (14). Flexor enthesopathy is radiologically characterized by the presence of one or more calcified bodies in the flexor muscles near the medial epicondyle or by a spur arising caudally at the medial epicondyle (11, 14).

HIF/IOHC and flexor enthesopathy may occur individually or in combination with ED disorders (7–9, 11, 15). These conditions are more common in males and frequently occur bilaterally (2, 6, 9, 11). Most of the dogs with HIF/IOHC, flexor enthesopathy and/or ED will develop elbow joint osteoarthritis (2, 8, 16). In addition to clinical examination, high-quality, well-positioned radiographs are essential for reliable ED diagnosis (17). Within the ED complex, UAP and OCD can typically be diagnosed accurately using radiography (2, 18). In contrast, MCPD is rarely visible directly on radiographs and is usually suspected based on secondary changes, requiring computed tomography (CT) for diagnostic confirmation (19–21). Although HIF/IOHC can largely be diagnosed by radiography, CT demonstrates greater sensitivity (7, 9). Radiological features of flexor enthesopathy include irregular margins of the medial humeral epicondyle, a spur on the caudodistal margin of the medial epicondyle (epicondylar spur), and variably distinct calcified bodies in the soft tissues surrounding the medial epicondyle (10, 15, 16). These calcifications may be located within the origin tendons of the flexor muscles, including M. pronator teres, M. flexor carpi radialis, M. flexor carpi ulnaris, M. flexor digitorum superficialis, and M. flexor digitorum profundus (15, 22–24). In some cases, calcified bodies are also associated with the joint capsule and/or the medial collateral ligament (15, 22, 23).

Not all forms of flexor enthesopathy can be clearly identified using radiographs, making CT examination necessary. CT allows visualization of irregular margins of the medial epicondyle with cortical sclerosis or thickening (epicondylar spur), thickening of the flexor muscles, and calcified bodies in the soft tissues of the medial epicondyle in the bone window (11, 12). Intravenous contrast administration can further demonstrate enhancement of the flexor muscles (11, 12).

As no previous studies have assessed the diagnostic value of intravenous contrast-enhanced CT in canine elbow disorders, the aim of this study was to address this gap.

## 2 Materials and methods

### 2.1 Study design

This retrospective study reviewed the database of the Clinic for Small Animal Surgery and Reproduction, Ludwig-Maximilian University, Munich, Germany, for dogs that underwent CT examinations of both elbow joints with intravenous contrast administration between May 2014 and December 2020. During this period, it was standard practice at the clinic to perform post-contrast imaging on every dog that underwent an elbow CT scan. Dogs with unilateral or bilateral lameness attributable to elbow joint disease were included, as confirmed by orthopedic examination and imaging. Diagnostic imaging consisted of radiographs of both elbows in at least two planes, as well as CT imaging of the elbow joints following intravenous contrast administration. Both elbow joints of the included dogs were evaluated. Depending on CT findings, arthroscopy was performed in selected cases. Overall, unilateral or bilateral arthroscopy was carried out in 85% of dogs, corresponding to 51% of all elbow joints. Absence of arthroscopy did not constitute an exclusion criterion. Exclusion criteria were as follows: lameness unrelated to the elbow joint, incomplete radiographs at the time of examination, or a history of previous surgery on one of the elbow joints. For each dog, breed, age, sex, and body weight were recorded. Standard mediolateral extended and craniocaudal radiographs of both elbow joints were obtained using a Siemens Axiom Luminos dRF scanner (Siemens Healthcare GmbH, Erlangen, Germany). In a small number of cases, only recent radiographs provided by the referring veterinarian were available. These images were deemed sufficient for reliable interpretation, and the corresponding dogs were included. Radiographs were assessed for signs of elbow joint disease or suspected disorders, and periarticular osteophytes were graded according to the guidelines of the International Elbow Working Group (IEWG) (Table 1) (25).

**Table 1:** Osteoarthritis classification of the international elbow working group (IEWG).

Osteoarthritis scoring	Radiographic findings
Normal elbow joint	No evidence of osteoarthritis
Mild osteoarthritis	Presence of osteophytes < 2 mm
Moderate osteoarthritis	Presence of osteophytes 2–5 mm
Severe osteoarthritis	Presence of osteophytes > 5 mm

Suspicion of MCPD was noted when present. An epicondylar spur was radiographically identified as an osteophytic growth on the distal edge of the medial epicondyle of the humerus. A caudal calcified body was defined as a calcification in the soft tissue caudodistal to the medial epicondyle, and a medial calcified body was defined as a calcification in the soft tissue mediodistal to the medial epicondyle.

CT imaging was performed under general anesthesia in all dogs using a CT SOMATOM Definition AS (Siemens Healthcare GmbH, Erlangen, Germany). The dogs were positioned in sternal recumbency with their elbows extended forward and the head bent to one side to avoid beam hardening artifacts. For each elbow, raw data were acquired with a slice thickness of 0.6 or 0.75 mm, a pitch of 0.8, and a rotation time of 1 s. The acquisition parameters were 120 kV and 350 mA. The contrast agent (2 mL/kg) Accupaque 300 (GE Healthcare Buchler GmbH & Co. KG, Braunschweig, Germany) was administered intravenously via the lateral saphenous vein using a fully automated contrast agent injector (MEDRAD Stellant; Bayer Vital GmbH, Bayer Healthcare Radiology, Leverkusen, Germany). The scan was repeated 120 s after administration using the soft-tissue algorithm. Scans were reconstructed using a 512 × 512 matrix. All slices were reviewed in the bone window (window center, 500 HU; window width, 2,500 HU) and in the soft-tissue window (window center, 40–80 HU; window width, 300–450 HU).

Unenhanced CT images were evaluated using a bone window to diagnose elbow joint disorders. MCPD was diagnosed if fragmentation or fissuring of the medial coronoid process was present and/or if demineralization and proliferation of the medial coronoid process were observed. An epicondylar spur was defined as an osteophytic growth on the distal edge of the medial epicondyle. A caudal calcified body was confirmed as a calcification in the soft tissue caudodistal to the medial epicondyle, and a medial calcified body as a calcification in the soft tissue mediodistal to the medial epicondyle. Classification of periarticular osteophytes was not repeated with CT, as Schubert et al. demonstrated that the severity of osteoarthritis is assessed identically on radiographs and CT scans (26). Furthermore, as illustrated above, the assessment of periarticular osteophytes in radiographs permitted grading in accordance with the International Elbow Working Group (IEWG) guidelines.

The final diagnosis of the elbow joint was based on radiographic evaluation, with confirmation by CT imaging in the bone window. Arthroscopic findings were considered when available.

The radiographic and CT images were evaluated by three veterinarians: a Diagnostic Imaging Specialist in Germany, a doctoral student and a Surgery and Small Animal Specialist in Germany who is an active member of the Society of X-ray Diagnosis of Genetically Influenced Skeletal Diseases in Small Animals (GRSK). The images were evaluated individually, after which the diagnoses were determined by a majority vote. These diagnoses included primary diseases and periarticular osteophytes. Possible primary diseases included MCPD, UAP, OCD, HIF/IOHC, epicondylar spur, caudal calcified bodies, and medial calcified bodies. One elbow joint could be diagnosed with multiple primary diseases and periarticular osteophytes.

**Figure 1:**
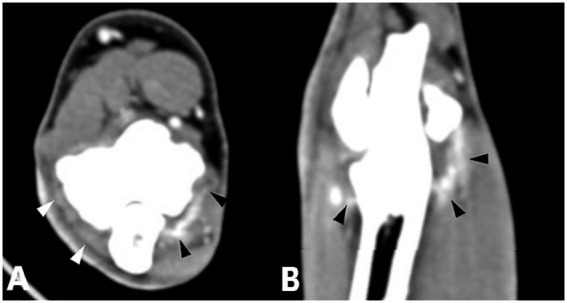
Computed tomography slices after contrast agent administration showing enhancement of the joint capsule. **(A)** Transverse CT slice of a right elbow joint with increased joint fluid and enhancement of the joint capsule (black and white arrowheads). **(B)** Dorsal reconstruction of the same elbow joint shown in **(A)** demonstrating increased joint fluid and enhancement of the joint capsule (black arrowheads).

**Figure 2:**
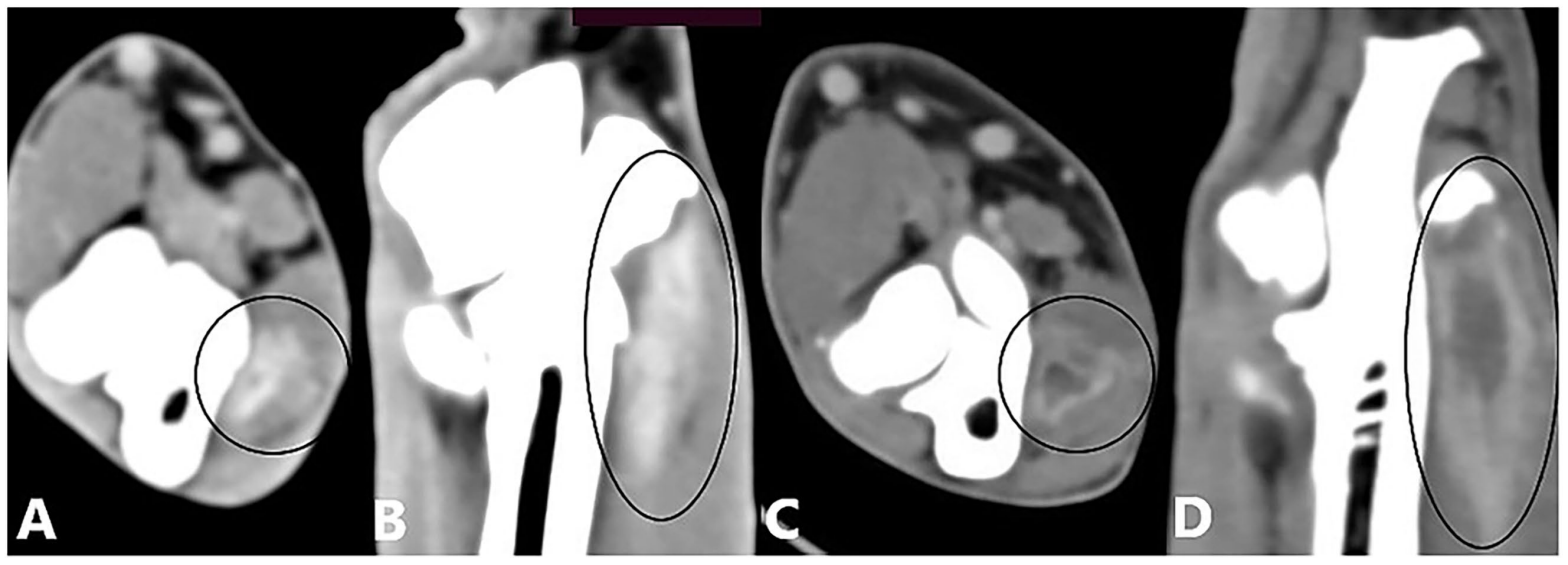
Computed tomography slices after contrast agent administration showing enhancement of the flexor muscles. **(A,B)** Transverse and dorsal CT slices of a right elbow joint showing a hyperattenuated zone (contrast enhancement) in the flexor muscles (black circle). **(C,D)** Transverse and dorsal CT slices of a right elbow joint showing enhancement of the flexor muscles, visible as a hypoattenuated center surrounded by a hyperattenuated rim (black circle).

CT images obtained after contrast administration were examined in the soft-tissue window to identify enhancement of the joint capsule, one or more flexor muscles, or their origin tendons. The degree of enhancement was not considered in the analysis. Enhancement of the joint capsule was defined as a hyperattenuated periarticular area (Figure 1). Enhancement of the flexor muscles was defined as a hyperattenuated area within the flexor muscles or their origin tendons, or as a hypoattenuated center surrounded by a hyperattenuated rim (Figure 2). Joint capsule and flexor muscle enhancement could occur simultaneously in a single joint. Because of the high sensitivity of CT imaging in detecting flexor enthesopathy, elbow joints with flexor muscle enhancement were considered to have flexor enthesopathy (12). The control group, used for comparison with diseased elbow joints, comprised the elbow joints of included dogs that showed no evidence of periarticular osteophytes on radiography or CT in the bone window, no lameness (contralateral joints), and no contrast enhancement.

### 2.2 Statistics

Statistical analysis was performed using IBM SPSS Statistics software, version 29.0.1. Initially, findings were descriptively processed by calculating both the absolute and relative incidences of individual findings, as well as combinations of findings. Contingency tables were created to assess whether contrast enhancement, the dependent variable, occurred disproportionately in relation to specific elbow joint pathologies. A chi-square test was used to calculate the probability of error (p) for binary trait manifestations. If the expected incidence in a cell of the contingency table was less than 10, Fisher’s exact test was applied. For the ordinal-scale variables, the error probability was calculated using the Somers’D method. In this approach, two groups were formed for each pathological feature (e.g., MCPD: yes/no) without considering additional findings. Additionally, groups with positive findings were compared to the control group in terms of enhancement patterns using the chi-square test, Fisher’s exact test, or Somers’ method. Supplementary multifactorial variance analyses were performed to identify the pathologies most likely associated with contrast enhancement. Because data dependency was present due to the inclusion of both elbow joints from many dogs and because the dependent variable also contained ordinal data, a generalized linear model for repeated measurements (GEE) was applied. The significance level was set at *p* < 0.05.

## 3 Results

### 3.1 Population

A total of 163 dogs (326 examined elbow joints) were included in this study. Of these, 82 were female and 81 were male. The mean age was 4.4 years (± 3.5 years) and the mean weight was 30 kg (± 11.1 kg). The most common breed was mixed-breed, accounting for 56 of 163 dogs (34%). Labrador Retrievers were the second most common, with 26 of 163 dogs (16%), followed by Rottweilers, with 9 of 163 dogs (5.5%). At the time of examination, 149 of 163 dogs (91.4%) exhibited unilateral lameness, and 14 dogs (8.6%) exhibited bilateral lameness.

### 3.2 Incidence of diagnoses based on the evaluation of radiography and unenhanced CT

Pathological findings were identified in 304 of 326 elbow joints (93.3%). One joint showed no detectable pathological findings on radiography or CT imaging in the bone window, although contrast enhancement of the joint capsule was also observed. Arthroscopy was not performed in this case, but the joint was included in the analysis. Thus, 305 elbow joints (93.6%) showed at least one pathological finding on imaging. Twenty-one elbow joints exhibited no notable diagnoses and were used as controls.

A total of 605 pathological findings were recorded across 305 elbow joints. Periarticular osteophytes was the most common condition, accounting for 274 of the 605 pathological findings (45.3%) regardless of the presence of a primary disease (Table 2).

**Table 2:** Incidence of diagnoses determined by radiography and unenhanced CT.

Diagnoses	Number (*n* = 605)	Percentage of all findings (%)
Periarticular osteophytes (all grades)	274	45.3%
MCPD	247	40.8%
UAP	3	0.5%
OCD	12	2.0%
HIF/IOHC	5	0.8%
Epicondylar spur	41	6.8%
Caudal calcified body	11	1.8%
Medial calcified body	12	2.0%

Of the 305 elbow joints examined, one primary disease was present in 214 of these joints (70.2%). Of these, 196 (91.5%) were diagnosed with MCPD and 8 (3.7%) with an epicondylar spur (Table 3).

**Table 3:** Elbow joints evaluated by radiography and unenhanced CT showing one primary disease (*n* = 214) and its incidence.

Primary diseases	Number (*n* = 214)	Percentage of the 214 elbow joints	Percentage of all 305 elbow joints
MCPD	196	91.5%	64.3%
UAP	3	1.4%	1%
OCD	1	0.5%	0.3%
HIF/IOHC	1	0.5%	0.3%
Epicondylar spur	8	3.7%	2.6%
Caudal calcified body	2	0.93%	0.7%
Medial calcified body	3	1.4%	1%

Of the 305 elbow joints analyzed, two primary diseases were diagnosed in 43 of these joints (14.1%). The most common combination was MCPD with an epicondylar spur, observed in 23 of the 43 joints (53.5%). The second most frequent combination was MCPD and OCD, present in 9 of the 43 joints (20.9%) (Table 4).

**Table 4:** Elbow joints evaluated by radiography and unenhanced CT showing two primary diseases (*n* = 43) and their incidence.

Primary diseases	Number (*n* = 43)	Percentage of the 43 elbow joints	Percentage of all 305 elbow joints
MCPD + epicondylar spur	23	53.5%	7.5%
Epicondylar spur + caudal calcified body	1	2.3%	0.3%
MCPD + OCD	9	20.9%	3%
MCPD + caudal calcified body	2	4.7%	0.7%
MCPD + HIF/IOHC	3	7%	1%
MCPD + medial calcified body	4	9.3%	1.3%
Epicondylar spur + medial calcified body	1	2.3%	0.3%

Of the 305 elbow joints evaluated, 10 (3.3%) had three or four primary diseases simultaneously. Of these, nine joints presented three primary diseases and one joint presented four. The most common combination of three primary diseases was MCPD, epicondylar spur, and caudal calcified body, observed in 4 of the 9 joints (44.4%) (Table 5).

**Table 5:** Elbow joints evaluated by radiography and unenhanced CT showing three or four primary diseases (*n* = 10) and their incidence.

Number of diseases	Primary diseases	Number (*n* = 10)	Percentage of the 10 elbow joints	Percentage of all 305 elbow joints
3	MCPD + epicondylar spur + caudal calcified body	4	40%	0.7%
3	MCPD + epicondylar spur + medial calcified body	2	20%	0.7%
3	MCPD + HIF/IOHC + epicondylar spur	1	10%	0.3%
3	MCPD + OCD + medial calcified body	1	10%	0.3%
3	MCPD + caudal calcified body + medial calcified body	1	10%	0.3%
4	MCPD + OCD + epicondylar spur + caudal calcified body	1	10%	0.3%

### 3.3 Incidences and locations of contrast enhancement

Contrast enhancement was observed in 137 elbow joints (44.9%). Of these, 94 joints (68.6%) showed enhancement of the joint capsule only, 16 joints (11.7%) showed enhancement of the flexor muscles only, and 27 joints (19.7%) showed enhancement of both the joint capsule and flexor muscles. No correlation could be established between age, divided into three groups (< 1 year, 1–3 years and > 3 years), and contrast enhancement. However, when age was divided into two groups (<1 year and >1 year), a significant correlation was found between the enhancement of the flexor muscles and age over 1 year (*p* = 0.026).

### 3.4 Correlation of the 605 diagnoses and contrast enhancement

Periarticular osteophytes, MCPD, epicondylar spur, and medial calcified body were disproportionately associated with enhancement of the joint capsule (Table 6). Multifactorial variance analysis indicated that periarticular osteophytes exerted the strongest influence on joint capsule enhancement. For enhancement of the flexor muscles, positive associations were observed with periarticular osteophytes, epicondylar spur, and caudal calcified body. In contrast, medial calcified bodies showed no correlation with flexor muscle enhancement (Table 6). Multifactorial variance analysis demonstrated that an epicondylar spur exerted the strongest influence on flexor muscle enhancement.

**Table 6:** Correlation of the 605 diagnoses with the contrast enhancement of the joint capsule and flexor muscles.

Diagnoses	Number (*n* = 605)	Enhancement of the joint capsule	Enhancement of the flexor muscles
Osteoarthritis of varying degrees	274	*p < 0.001	*p = 0.002
MCPD	247	*p < 0.001	-*p* = 0.283
UAP	3	-*p* = 0.055	-*p* = 1.000
OCD	12	-*p* = 0.360	-*p* = 0.475
HIF/IOHC	5	-*p* = 0.123	-*p* = 0.612
Epicondylar spur	41	*p < 0.001	*p < 0.001
Caudal calcified body	11	-*p* = 0.421	*p = 0.002
Medial calcified body	12	*p = 0.030	-*p* = 0.707

*Significant correlation (*p* < 0.05). -No significant correlation (*p* ≥ 0.05).

### 3.5 Correlation of elbow joints with one (*n* = 214), two (*n* = 43), three (*n* = 9), or four (*n* = 1) primary diseases and the contrast enhancement of the joint capsule and flexor muscles compared with the control group (*n* = 21)

**Figure 3:**
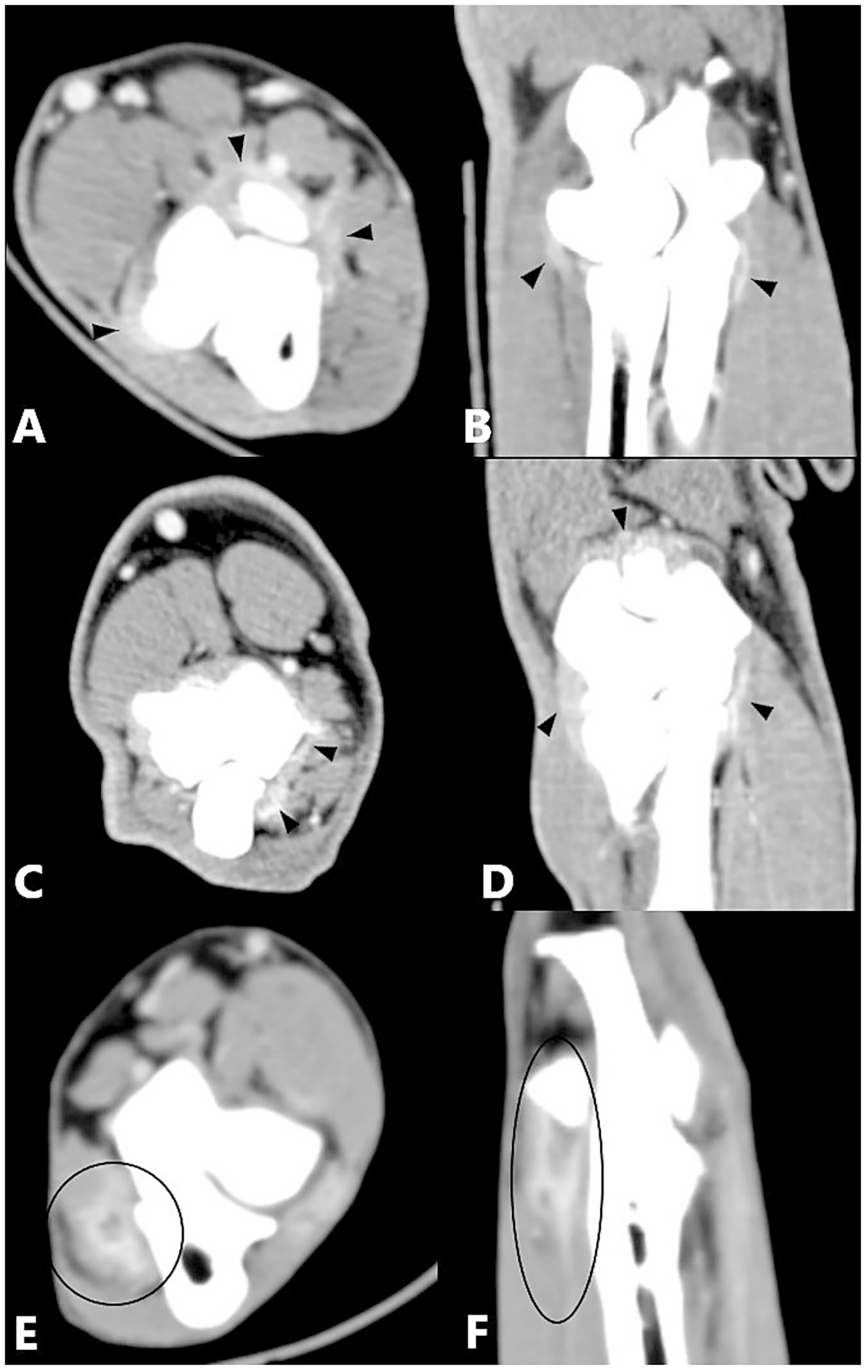
Computed tomography slices of elbow joints with a single primary disease showing significant correlation with contrast enhancement of the joint capsule or flexor muscles. **(A,B)** Left elbow joint with MCPD showing contrast enhancement of the joint capsule (black arrowheads). **(C,D)** Right elbow joint with UAP showing contrast enhancement of the joint capsule (black arrowheads). **(E,F)** Left elbow joint with an epicondylar spur showing contrast enhancement of the flexor muscles (black circle).

The primary diseases described below generally included additional periarticular osteophytes, which is not listed separately. Periarticular osteophytes were analyzed separately. There was a significant correlation between MCPD (*n* = 196), UAP (*n* = 3), and enhancement of the joint capsule compared with the control group. An epicondylar spur (*n* = 8) showed a significant correlation with flexor muscle enhancement compared with the control group (Figure 3). No significant difference from the control group was observed for other single primary diseases and no statistical significance could be calculated where enhancement was absent or case numbers were insufficient (Table 7).

**Table 7:** Correlation of elbow joints with one primary disease (*n* = 214) and the contrast enhancement of the joint capsule and flexor muscles compared to the control group (*n* = 21).

Primary diseases compared to the control group	Number (*n* = 214)	Enhancement of the joint capsule	Enhancement of the flexor muscles
MCPD	196	*p < 0.001	- *p* = 0.395
UAP	3	*p < 0.001	x
OCD	1	x	x
HIF/IOHC	1	x	x
Epicondylar spur	8	x	*p < 0.001
Caudal calcified body	2	x	- *p* = 0.087
Medial calcified body	3	- *p* = 0.125	x

*Significant difference from the control group (*p* < 0.05). - No significant difference from the control group (*p* ≥ 0.05). x No evaluation due to lack of enhancement. # No significance to calculate due to *n* = 1.

**Figure 4:**
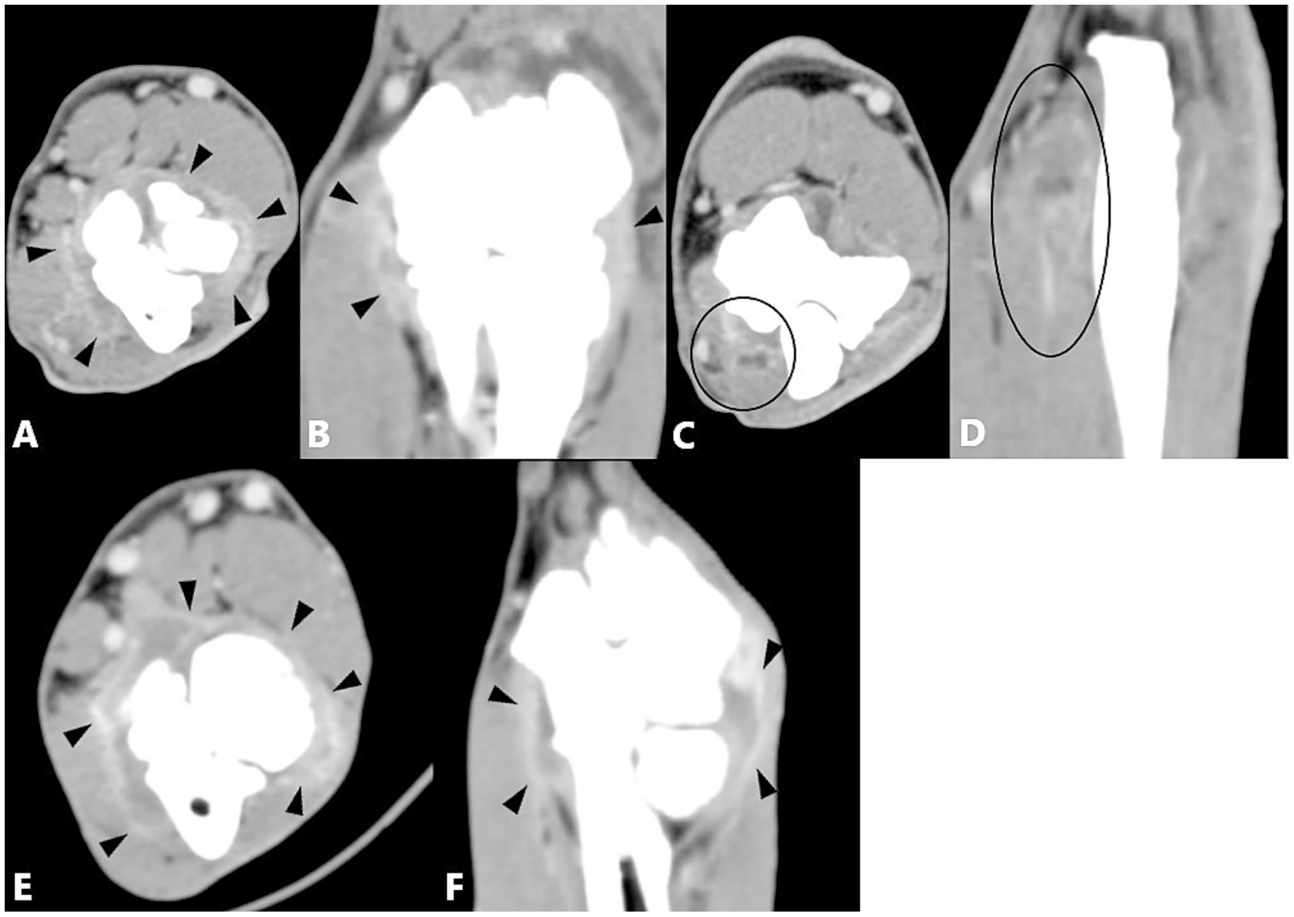
Computed tomography slices of elbow joints with two primary diseases showing significant correlation with contrast enhancement of the joint capsule and/or flexor muscles. **(A,B)** Left elbow joint with MCPD and epicondylar spur showing joint fluid and contrast enhancement of the joint capsule (black arrowheads). **(C,D)** Same joint showing contrast enhancement of the flexor muscles (black circle). **(E,F)** Left elbow joint with MCPD and medial calcified body showing joint fluid and contrast enhancement of the joint capsule (black arrowheads).

The combination of MCPD and an epicondylar spur (*n* = 23) produced significantly greater enhancement of the joint capsule and flexor muscles compared with the control group. A significant correlation was also observed between joint capsule enhancement and the combination of MCPD and a medial calcified body (*n* = 4) compared with the control group (Figure 4). No significant difference from the control group was observed for other combinations of two primary diseases, and no statistical significance could be calculated when enhancement was absent or case numbers were insufficient (Table 8).

**Table 8:** Correlation of elbow joints with two primary diseases (*n* = 43) and the contrast enhancement of the joint capsule and flexor muscles compared to the control group (*n* = 21).

Combination of primary diseases compared to the control group	Number (*n* = 43)	Enhancement of the joint capsule	Enhancement of the flexor muscles
MCPD + epicondylar spur	23	*p < 0.001	*p < 0.001
Epicondylar spur + caudal calcified body	1	x	#
MCPD + OCD	9	- *p* = 0.005	x
MCPD + caudal calcified body	2	- *p* = 0.087	x
MCPD + HIF/IOHC	3	x	x
MCPD + medial calcified body	4	*p = 0.002	x
Epicondylar spur + medial calcified body	1	#	x

*Significant difference from the control group (*p* < 0.05). - No significant difference from the control group (*p* ≥ 0.05). x No evaluation due to lack of enhancement # No significance to calculate due to *n* = 1.

**Figure 5:**
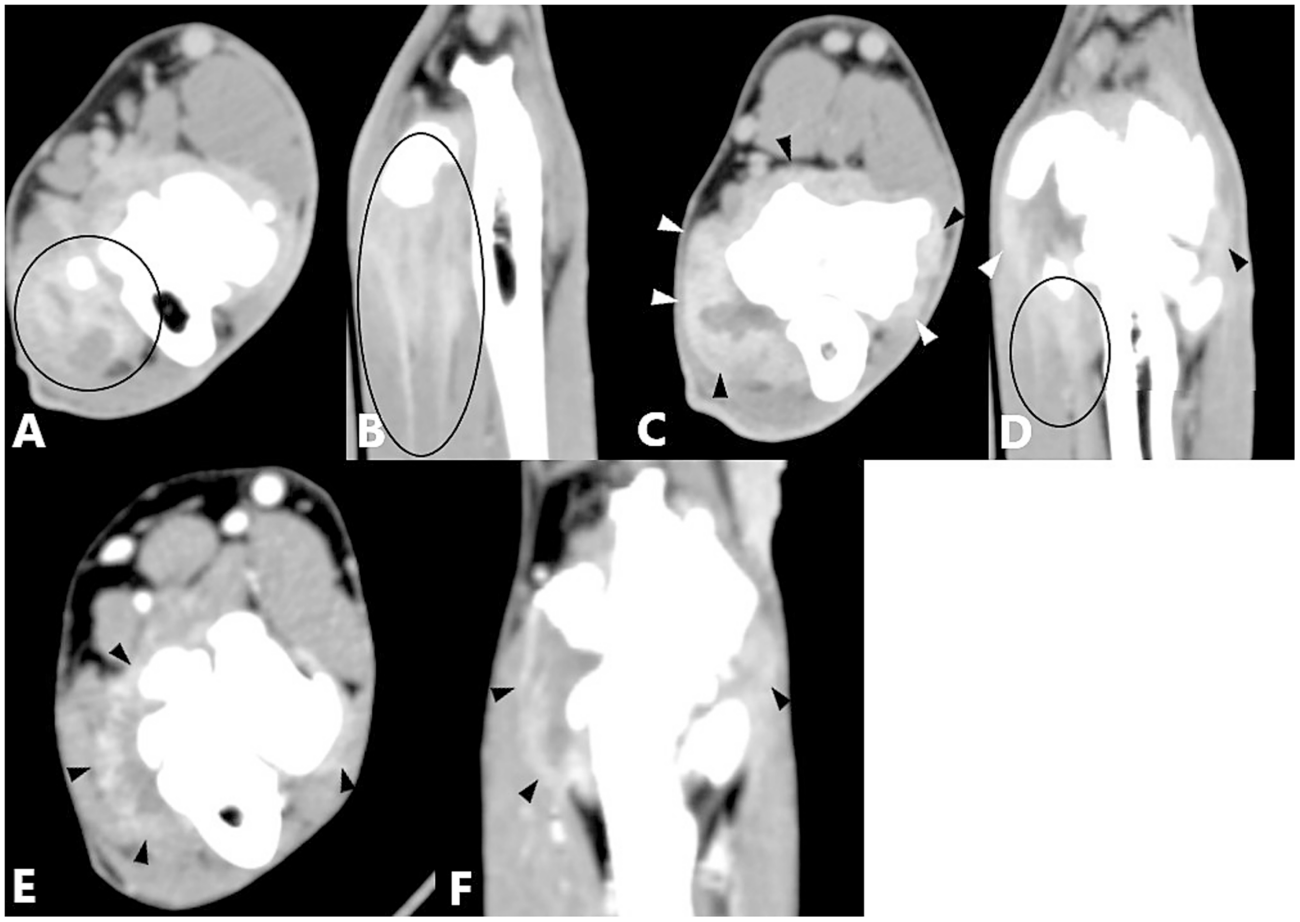
Computed tomography slices of elbow joints with three primary diseases showing significant correlation with contrast enhancement of the joint capsule and/or flexor muscles. **(A,B)** Left elbow joint with MCPD, epicondylar spur, and a caudal calcified body showing contrast enhancement of the flexor muscles (black circle). **(C,D)** Same joint showing increased joint fluid and contrast enhancement of the joint capsule (black and white arrowheads). The dorsal reconstruction **(D)** also shows contrast enhancement of the flexor muscles (black circle). **(E,F)** Left elbow joint with MCPD, epicondylar spur, and a medial calcified body showing joint fluid and contrast enhancement of the joint capsule (black arrow heads).

Among the three primary diseases combinations, those involving MCPD, an epicondylar spur, and a caudal calcified body (*n* = 4), or MCPD, an epicondylar spur, and a medial calcified body (*n* = 2), showed a correlation with joint capsule enhancement compared with the control group. The combination of MCPD, an epicondylar spur, and a caudal calcified body also showed a significant association with flexor muscle enhancement compared with the control group (Figure 5). No significant differences were observed for other three primary diseases combinations, either due to the absence of enhancement or insufficient case numbers. Enhancement of both the joint capsule and flexor muscles was observed in a single elbow joint with four primary diseases, but statistical significance could not be determined (Table 9).

**Table 9:** Correlation of elbow joints with three or four primary diseases (*n* = 10) and the contrast enhancement of the joint capsule and flexor muscles compared to the control group (*n* = 21).

Number of primary diseases	Combination of primary diseases compared to the control group	Number (*n* = 10)	Enhancement of the joint capsule	Enhancement of the flexor muscles
3	MCPD + epicondylar spur + caudal calcified body	4	*p = 0.002	*p = 0.002
3	MCPD + epicondylar spur + medial calcified body	2	*p = 0.004	- *p* = 0.087
3	MCPD + HIF/IOHC + epicondylar spur	1	x	x
3	MCPD + OCD + medial calcified body	1	#	x
3	MCPD + caudal calcified body + medial calcified body	1	x	x
4	MCPD + OCD + epicondylar spur + caudal calcified body	1	#	#

*Significant difference from the control group (*p* < 0.05). - No significant difference from the control group (*p* ≥ 0.05). x No evaluation due to lack of enhancement. # No significance to calculate due to *n* = 1.

### 3.6 Enhancement of the flexor muscles depending on features of flexor enthesopathy, regardless of other diagnoses

Among elbow joints with an epicondylar spur but no calcified bodies (*n* = 32), 22 (68.8%) showed flexor muscle enhancement. Of the elbow joints with a caudal calcified body but no epicondylar spur (*n* = 4), only one exhibited flexor muscle enhancement. No enhancement of the flexor muscles was observed in any of the elbow joints with a medial calcified body but no epicondylar spur (*n* = 8).

The combination of an epicondylar spur and a caudal calcified body (*n* = 6) was associated with flexor muscle enhancement in five cases. Among the elbow joints with an epicondylar spur and a medial calcified body (*n* = 3), enhancement of the flexor muscles was observed in one joint. The combination of medial and caudal calcified bodies occurred only once; however, this case did not demonstrate flexor muscle enhancement.

Of the 43 elbow joints showing flexor muscle enhancement, 14 (32.6%) did not display an epicondylar spur, caudal calcified body, or medial calcified body. Of these, three (21.4%) exhibited periarticular osteophytes alone, while 11 (78.6%) exhibited MCPD alongside periarticular osteophytes.

### 3.7 No primary disease on radiographs and unenhanced CT

After examination of radiographs and unenhanced CT scans of 326 elbow joints, no primary disease was diagnosed in 59 joints (18.1%). Twenty-one joints showed no diagnoses or enhancement and served as the control group. Thirty-seven elbow joints showed periarticular osteophytes only. Of these, six (16.2%) showed joint capsule enhancement, and three (8.1%) showed flexor muscle enhancement. Additionally, one elbow joint showed no abnormalities on radiographs or unenhanced CT scans but showed enhancement of the joint capsule. In summary, contrast enhancement was observed in 10 of the 59 joints (16.9%) that had not been diagnosed a primary disease by radiography or unenhanced CT.

### 3.8 Periarticular osteophytes

Of the 274 elbow joints with periarticular osteophytes, 109 (39.8%), 138 (50.4%), and 27 (9.9%) were classified as mild, moderate, and severe osteoarthritis, respectively.

Elbow joints with a single primary disease of MCPD, an epicondylar spur, a medial calcified body, a combination of MCPD and OCD, or MCPD and a medial calcified body most often demonstrated moderate periarticular osteophytes. Above-average rates of severe periarticular osteophytes were observed in the combinations of MCPD with an epicondylar spur (*n* = 23) and MCPD with an epicondylar spur and caudal calcified body (*n* = 4).

In general, the greater the number of primary diseases in the elbow joint, the greater the likelihood of severe periarticular osteophytes (*p* < 0.001). As periarticular osteophytes severity increased, the incidence of enhancement of both the joint capsule and flexor muscles also increased (*p* < 0.001).

### 3.9 Undesirable side effects of contrast agent

Of the 163 dogs that received contrast, one developed sinus arrhythmia accompanied by extrasystoles and respiratory distress despite low CO₂ levels under anesthesia. The dog’s condition stabilized rapidly when 100% oxygen was administered.

## 4 Discussion

Previous studies have not addressed the significance of contrast administration in CT examinations of the elbow joint for various diseases. Therefore, this study aimed to evaluate the diagnostic value of contrast-enhanced CT imaging of the elbow joint under various conditions.

This study found that contrast enhancement was most commonly observed in the joint capsules. The literature currently offers no description of joint capsule enhancement, making comparisons in this regard impossible. A correlation was observed between MCPD, UAP, and joint capsule enhancement compared with the control group. It is assumed that active inflammation and synovitis resulting from these diseases caused the enhancement. The literature describes synovitis in conditions such as MCPD, OCD, UAP, flexor enthesopathy, and secondary osteoarthritis (2, 13, 27–29). In this study, joint capsule enhancement was most strongly influenced by periarticular osteophytes. This suggests that elbow joints exhibiting joint capsule enhancement are highly likely to show an active synovitis as part of the osteoarthritis progression. Additionally, the incidence of joint capsule enhancement increased with increasing periarticular osteophytes severity. Because osteophytes develop in response to inflammation (30) it can be inferred that increased inflammation may lead to greater osteophyte formation. Joint capsule enhancement was assessed in both elbows of the same dog. Future studies should investigate whether joint capsule enhancement is present in affected elbow joints without concurrent lameness.

Enhancement of both the joint capsule and flexor muscles indicated the presence of concomitant flexor enthesopathy. Contrast-enhanced evaluation is helpful in distinguishing between primary and concomitant flexor enthesopathy. This distinction is important because the recommended treatments for these conditions differ (11, 16). The study confirmed that concomitant flexor enthesopathy occurs more frequently than primary flexor enthesopathy, as simultaneous joint capsule and flexor enhancement occurred more often than isolated flexor enhancement (11). Therefore, even if MCPD, UAP, OCD, or HIF/IOHC is diagnosed, a contrast agent should be administered to avoid missing concomitant flexor enthesopathy.

In this study, the strongest correlation was found between flexor enhancement and an epicondylar spur. Other studies have also identified the epicondylar spur as a common feature of flexor enthesopathy (10, 16). Furthermore, it was established that the severity of periarticular osteophytes correlates with the incidence of flexor enhancement. This suggests that a higher degree of periarticular osteophytes may be present when active flexor enthesopathy is evident in the elbow joint, or that flexor enthesopathy develops as a result of osteoarthritis. According to De Bakker et al., elbow joints affected by periarticular osteophytes and pathologies of the flexor muscles and their origins can be described as concomitant flexor enthesopathy (16). Further research is required to confirm this hypothesis.

Flexor enhancement occurred significantly more frequently in elbow joints diagnosed with an epicondylar spur. No other single primary disease showed a correlation with contrast enhancement of the flexor muscles, indicating that it was specifically associated with an epicondylar spur.

In the present study, one-third of the elbow joints with an epicondylar spur but no additional calcified body showed no enhancement of the flexor muscles. Elbow joints with a caudal calcified body but no epicondylar spur displayed enhancement of the flexor muscles in three of four cases. The presence of both an epicondylar spur and a caudal calcified body resulted in no enhancement of the flexor muscles in one of six cases. Because detailed breakdowns of flexor enhancement associated with epicondylar spurs and/or medial or caudal calcified body findings are not available in the literature, no comparison can be made in this regard. Flexor enhancement indicates increased blood flow due to repair processes in the flexor muscles and their origin tendons (31). However, in the specified elbow joints, no increased blood flow accompanied by flexor enhancement was evident, suggesting that flexor enthesopathy was inactive. This may be because the disease was at a late stage and involved tendon degeneration without inflammation, which is similar to what has been described in human medicine for medial epicondylitis (32). This suggests that contrast-enhanced imaging could help distinguish between active and inactive flexor enthesopathy. However, further studies are required to confirm this hypothesis, given the small sample size of the present study.

Investigations into flexor enhancement revealed that none of the cases involving a medial calcified body without an epicondylar spur exhibited flexor muscle enhancement. This finding suggests that a medial calcified body is unlikely to be associated with flexor enhancement or active flexor enthesopathy. This aligns with earlier studies suggesting that calcified bodies in the medial epicondyle may be asymptomatic incidental findings (11, 33). Meyer-Lindenberg et al. also described calcified bodies at the medial epicondyle as a rare cause of lameness; however, they did not examine the presence of an epicondylar spur (15). The distinction between medial and caudal calcified bodies and their correlation with flexor enhancement has not been addressed in the available literature and therefore requires further research.

Of the 43 elbow joints exhibiting contrast enhancement of the flexor muscles, 14 showed no evidence of an epicondylar spur or calcified body, either medial or caudal. These elbow joints were likely in the early stage of flexor enthesopathy, affecting only the soft tissues (10, 12, 34). This early stage could be detected through contrast administration in this study as well as in others (10, 12). Therefore, a contrast agent should be administered during CT examination of the elbow joints, even in the absence of bony signs of flexor enthesopathy.

The most frequent finding in this study was periarticular osteophytes of varying degrees, which may be attributed to the fact that most elbow disorders lead to secondary osteoarthritis (2, 8, 16) The frequent occurrence of moderate periarticular osteophytes confirmed the observation of Komsta et al., who reported that most dogs presented with moderate osteoarthritis in at least one joint (5). In this study the diagnoses of MCPD, epicondylar spur, and medial calcified body, as well as the combinations MCPD with OCD and MCPD with medial calcified body, most often demonstrated moderate periarticular osteophytes. The combinations of MCPD with epicondylar spur, and MCPD with epicondylar spur and caudal calcified body, exhibited above-average severity of periarticular osteophytes.

Similarly, Komsta et al. observed moderate osteoarthritis in the elbow joints of dogs with ED (5). The relationship between flexor enthesiopathy and osteoarthritis has been studied by De Bakker et al., who found that primary flexor enthesiopathy is most typically associated with mild or moderate degree of osteoarthritis and concomitant flexor enthesiopathy exhibits more severe degrees of osteoarthritis (10–12, 16). The present study also found that the greater the number of diagnoses in a joint, the more frequently severe periarticular osteophytes was observed.

One contralateral elbow joint without lameness showed no radiographic or CT findings in the bone window; however, joint capsule enhancement was evident. The lame limb of the same dog displayed UAP. Therefore, overloading on the contralateral side may have caused joint capsule enhancement.

After evaluating radiography and unenhanced CT, it was found that 37 elbow joints only showed periarticular osteophytes. Nine of these joints exhibited contrast enhancement. Contrast enhancement was observed in 16.9% of elbow joints without a diagnosed primary disease after radiographic and unenhanced CT examinations. Therefore, a contrast agent should be administered to elbow joints for which a primary disease cannot be identified using radiography and unenhanced CT scans.

One of the 163 dogs exhibited adverse reactions to the contrast agent. This confirmed that contrast administration can lead to hypersensitivity in individual cases (35). Because extrasystoles occurred, this case can be classified as a more serious side effect, occurring in only 0.8% of the cases (35, 36). Cardiac dysfunction following contrast administration can lead to cardiac arrest (35). Contrast-induced nephropathy is a consequence of contrast agent administration that is described in the literature and should not be underestimated, even if it cannot be confirmed in the present study due to lack of information (37). However, the retrospective nature of the study means that side effects may be underrepresented due to incomplete documentation.

Further limitations of this study include the small number of cases involving certain combinations of primary diseases and the absence of arthroscopy in some elbow joints to confirm the findings.

The results of this study showed that additional contrast administration during CT imaging was not beneficial for diagnosing MCPD, OCD, UAP, or HIF/IOHC. Similarly, De Bakker et al. recommended contrast-enhanced CT only when lesions other than those of the ED complex are suspected (12). However, contrast enhancement revealed active inflammation and concomitant flexor enthesopathy. Overall, the administration of additional contrast in CT imaging was useful for flexor enthesopathy, as flexor muscle enhancement was specific and allowed the identification of flexor enthesopathy without epicondylar spur or calcified bodies.

In conclusion, a contrast agent could be administered during most CT scans of the elbow joint to ensure that no pathological changes are overlooked. However, the decision to administer a contrast agent should be made on an individual basis. It is important to weigh up the benefits for the treatment and prognosis against the risks of administering a contrast agent to the patient.

Another finding of this study suggests that a medial calcified body is probably not associated with contrast enhancement of the flexor muscles and, consequently, does not appear to belong to the flexor enthesopathy complex. However, this hypothesis warrants further investigation.

## Data Availability

The raw data supporting the conclusions of this article will be made available by the authors, without undue reservation.
